# Greater target or lure variability? An exploration on the effects of stimulus types and memory paradigms

**DOI:** 10.3758/s13421-023-01483-7

**Published:** 2023-12-04

**Authors:** Haomin Chen, Andrew Heathcote, James D. Sauer, Matthew A. Palmer, Adam F. Osth

**Affiliations:** 1 University of Melbourne, Melbourne, Australia; 2 University of Amsterdam, Amsterdam, Netherlands; 3 University of Tasmania, Tasmania, Australia

**Keywords:** Recognition memory, Signal detection, Evidence variability, ROC

## Abstract

In recognition memory, the variance of the target distribution is almost universally found to be greater than that of the lure distribution. However, these estimates commonly come from long-term memory paradigms where words are used as stimuli. Two exceptions to this rule have found evidence for greater lure variability: a short-term memory task (Yotsumoto et al., *Memory & Cognition,* *36*, 282–294 2008) and in an eyewitness memory paradigm (Wixted et al., *Cognitive Psychology,* *105*, 81–114 2018). In the present work, we conducted a series of recognition memory experiments using different stimulus (faces vs. words) along with different paradigms (long-term vs. short-term paradigms) to evaluate whether either of these conditions would result in greater variability in lure items. Greater target variability was observed across stimulus types and memory paradigms. This suggests that factors other than stimuli and retention interval might be responsible for cases where variability is less for targets than lures.

Signal detection theory (SDT) is arguably the most influential framework for modeling how decisions are made in recognition memory tasks. According to SDT, memory strengths of targets and lures are represented by two separated but overlapping Gaussian distributions. A decision criterion is placed somewhere along the continuum of memory strength; an ‘old’ response is made if any test item generates a strength exceeding the criterion, otherwise a ‘new’ response is made (as illustrated in Fig. 1A). Predictions of SDT models are commonly tested via analyses of the empirical receiver operating characteristic (ROC) curve. ROCs are constructed by plotting the hit rate (HR) against the false alarm rate (FAR) across different levels of bias. Although bias can be manipulated in various ways such as manipulations on target proportions or payoffs (e.g., Dube & Rotello, 2012), a typical way to obtain an ROC is by plotting cumulative confidence ratings for hits and false alarms.

**Fig. 1:**
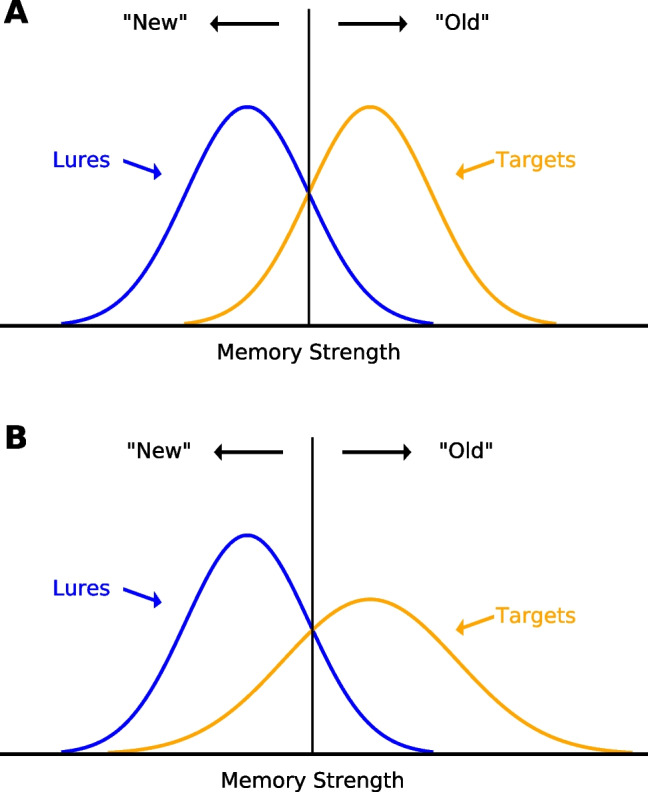
Illustration of the equal-variance (**A**) and unequal-variance SDT (**B**) models of recognition memory. *Note.* Memory strengths of targets and lures are represented by the bell-shaped curves. Decision criterion is represented by the *vertical line*. Signals exceeding the criterion generate an ‘old’ response, otherwise a ‘new’ response is generated

Gaussian SDT models predict a curvilinear ROC, as well as a linear z-transformed ROC (zROC). The slope of the zROC provides an estimate for the ratio of the standard deviation (SD) of the lure distribution to that of the target distribution ($\sigma _{lure}/\sigma _{target}$). If equal variability between targets and lures is assumed, a zROC slope of 1.0 is predicted; whereas if target variability exceeds lure variability, a zROC slope less than 1.0 will be predicted. Over the past decades, most if not all ROC studies in the field of recognition memory have reported zROC slopes less than 1.0, with a common value of approximately 0.80 (Benjamin, Diaz, & Wee, 2009; DeCarlo, 2007; Dube & Rotello, 2012; Glanzer & Adams, 1990; Glanzer, Hilford, Kim, & Adams, 1999; Glanzer, Kim, Hilford, & Adams, 1999; Heathcote, 2003; Hirshman & Hostetter, 2000; Kellen, Winiger, Dunn, & Singmann, 2021; Osth, Bora, Dennis, & Heathcote, 2017; Osth, Fox, McKague, Heathcote, & Dennis, 2018; Ratcliff, McKoon, & Tindall, 1994; Ratcliff, Sheu, & Gronlund, 1992; Wixted, 2007; Yonelinas, 1994). Following the SDT account, the SD of the target distribution is therefore about 1.25 (1/0.80) times that of the lure distribution, as shown in Fig. 1B. Such target–lure ROC asymmetry (i.e., the slope) has generally been found to be constant across a range of experimental manipulations that in principle should affect accuracy – this includes presentation time, level of attention and the number of presentations, although words of lower natural language frequency have generally been found to produce lower slopes than high frequency words (e.g., Ratcliff et al. , 1992; Ratcliff et al. , 1994; Glanzer et al. , 1999; Spanton & Berry, 2020). These findings have led to the proposition of the ’constancy-of-slopes’ generalization (Ratcliff et al., 1994). Although this generalization was later overturned by work showing that the ROC asymmetry can vary depending on task parameters or conditions (e.g., Heathcote , 2003; Meyer-Grant & Klauer, 2023; Dobbins , 2023; Hintzman , 2004), most of these manipulations failed to produce estimates of lure variability that exceed target variability.

Wixted (2007) provided the most common interpretation of the greater variability of targets, that encoding variability leads to different amounts of strength being added to each item during study, resulting in greater variability in target distribution. It is also important to note that several global matching models of recognition memory (see Clark & Gronlund, 1996; Osth & Dennis, 2020, for reviews) also made the a priori prediction of greater variability of targets, including the Minerva 2 (Hintzman, 1988), SAM (Gillund & Shiffrin, 1984), and REM (Shiffrin & Steyvers, 1997) models. In each of these models, the variability in memory strength scales with the mean memory strength. While in some cases this has led to an incorrect prediction that the zROC slope should decrease considerably in conditions of higher performance (e.g., Ratcliff et al. , 1992; Ratcliff et al. , 1994), models such as REM produced zROC slopes that roughly accorded with the data (Shiffrin & Steyvers, 1997). More recently developed global matching models such as the Osth and Dennis (2015) and Cox and Shiffrin (2017) models also make the prediction of greater target variability for similar reasons.

However, it should be mentioned that the majority of investigations that have found greater target variability did so under very particular conditions, namely that they used words as stimuli and employed long-term memory paradigms. Greater target variability is not guaranteed to be generalizable to other conditions. Indeed, a couple of exceptions to the finding of greater target variability have been found.

The most noteworthy example of the finding of greater lure variability comes from eyewitness memory paradigms. Wixted et al. (2018) applied three competing SDT models to the simultaneous lineup procedure, where participants viewed all members of a lineup including the suspect (i.e., photographed face of the actor from a mock crime video) and fillers (i.e., description-matched photographs of real human faces) at once. The best-fitting model parameters revealed the opposite of the usual pattern: greater *lure* variability. Parallel results were also reported by Wilson, Donnelly, Christenfeld, and Wixted (2019) and Dunn, Kaesler, and Semmler (2022), where in a sequential lineup procedure, participants make decisions about each lineup member individually. This resembles the method of eliciting old-new judgments in laboratory-based studies that have found evidence for greater target variability with word stimuli.

Another finding of greater lure variability came from a short-term memory task. Yotsumoto et al. (2008) investigated item recognition in a Sternberg recognition task (Sternberg, 1966). In this study, participants first viewed a short list of study items (sinusoidal gratings) and then were tested on their memory for a single test probe, which was either a target or a lure. The results of their ROC analyses demonstrated zROC slopes larger than 1.0 (1.1–1.3), which implied that the memory strengths of the lures are more variable than that of the targets.

It is therefore interesting and theoretically important to understand what is driving the divergent outcomes in the ratio of lure-to-target variability. A potential explanation for the reversal of the usual observed pattern in slope could be attributed to the differences in stimuli used across studies. Specifically, while studies that found slopes less than 1.0 almost exclusively adopted words as stimuli, studies that reported slopes larger than 1.0 have used stimuli that are non-linguistic (i.e., faces and sinusoidal luminance gratings). While there is no obvious theoretical explanation for why non-linguistic stimuli would elicit greater lure variability, a peripheral piece of evidence that supports a difference in stimulus types comes from studies that showed different effects of word and non-linguistic stimuli on the shape of the zROC curves – studies using word stimuli generally observed linear zROCs (e.g., Glanzer et al. , 1999), whereas studies using non-linguistic stimuli such as travel scenes and odors found curvilinear zROCs (Onyper, Zhang, & Howard, 2010; Howard, Bessette-Symons, Zhang, & Hoyer, 2006; Sherman, Atri, Hasselmo, Stern, & Howard, 2003; Fortin, Wright, & Eichenbaum, 2004). Findings like these suggest a possibility that the difference in stimulus types may be responsible for the divergent outcomes in previous ROC analyses of lure-to-target variability.

Another factor that is possibly responsible is the type of memory paradigm. Studies that have found greater target variability have typically used a long-term memory paradigm with a study-test method – in addition to studying a list of multiple items, participants are presented with a test list with multiple targets and lures. Eyewitness memory paradigms typically share the long-term memory component if there is a sufficiently long study-test delay, but invoke only a single studied item rather than a list and the test list contains a single target and a number of lures (usually five). The Yotsumoto et al. (2008) study used a short-term memory task with a short study list (three items) and a single test probe. Thus, it is possible that tests of short-term memory, short study lists, or short test lists may result in greater lure variability.

A theoretical explanation for why short study lists may induce greater lure variability comes from exemplar models of recognition memory (e.g., Kahana & Sekuler, 2002; Nosofsky , 1991; Osth, Zhou, Lilburn, & Little, 2023). Exemplar models use a global matching retrieval mechanism similar to the aforementioned REM and SAM models, but the similarity calculation is based on an exponential transformation of distance. This similarity calculation is consequential because the distance between a probe item and its own representation in memory (the self-match) is always 0, meaning there is no variability associated with it, while the distance between a probe item and other representations in memory is always variable. This means that in a short list of items, lure probes have *L* variable matches to the memory set, whereas target trials have *L - 1* variable matches. Consequently, lures have higher variability (Yotsumoto et al., 2008), and the difference should be more pronounced as *L* is decreased. The prediction of greater lure variability does not apply to models such as REM and SAM, where the match between a probe and its own representation in memory is not only variable, but often has greater variability than the match between a probe and other items in memory (Osth & Dennis, 2020).

We sought to clarify the conditions under which greater lure variability may be found. In particular, we manipulated different types of stimuli (images of faces vs. single words) across different memory paradigms (long-term memory using the study-test method vs. a short-term memory Sternberg paradigm) to evaluate whether either of these conditions would induce zROC slopes larger than 1.0, which are indicative of greater lure variability. All experiments compared faces and single words. A diagram of the basic procedures for our three experiments can be found in Fig. 2. In Experiment 1, a standard list memory paradigm is adopted, with lists of 24 study items. In Experiment 2a and 2b, Sternberg-styled procedures (Sternberg, 1966) were employed, in which a short series of study items (six and three items for Experiment 2a and 2b, respectively) were briefly presented and almost immediately followed by a single test probe item. As word stimuli are much more memorizable than face stimuli, especially in shorter lists, presentation time for words was shortened to better equate the performance between words and faces.

**Fig. 2:**
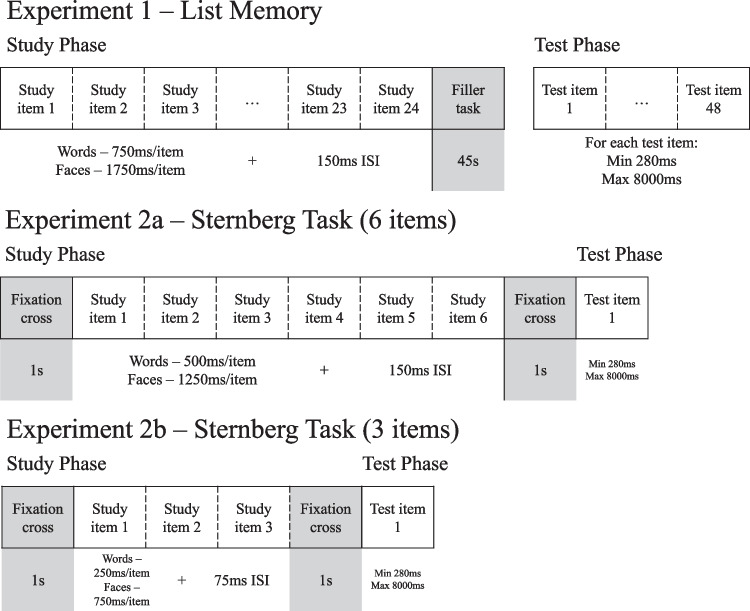
Diagram of the experimental procedures in Experiment 1, Experiment 2a, and Experiment 2b

In the following sections, we begin by setting up the exposition of each experiment, followed by detailed procedures described in the Method section. We then outline the results from our statistical and hierarchical Bayesian SDT analyses of the ROC data. To foreshadow our results, we did not find evidence for any reversal of the usual pattern, namely zROC slopes less than 1.0 or greater target variability, with stimulus types (faces and words) or tasks (long-term and short-term paradigms). Instead, greater target variability was found in all cases, although the ratio of target-to-lure-variability changed somewhat across stimuli and conditions, which also adds to evidence rejecting Ratcliff et al. (1994) ’constancy-of-slopes’ generalization.

## Experiment 1

Experiment 1 contrasted faces and words in a study-test long-term memory paradigm. The stimuli were manipulated across lists such that lists were comprised entirely of one stimulus type. To roughly equate the performance between faces and words, we employed longer presentation times for the faces condition than for the words condition.

### Method

#### Participants

Eighty participants were recruited. All participants were undergraduate students from the University of Melbourne who participated for course credits. The number of participants was selected in accordance with previous studies (e.g., Heathcote , 2003; Spanton & Berry, 2022), where 64–75 participants were recruited. It is important to note that in ROC studies, the number of trials per participant is likely to be more consequential than the number of participants. This is because large numbers of observations are required to obtain stable estimates of the zROC slope, which we are attempting to do for each individual participant. In the present experiment, each participant was tested on 768 trials per condition that were distributed across two 1-h sessions, which should be sufficient when comparing to 480 trials per participant in previous studies (e.g., Spanton & Berry, 2022). The study was approved by The University of Melbourne Psychological Sciences Human Ethics Advisory Group (Ethics ID: 12033). Informed consent was obtained from all participants.

While nine out of 80 participants only completed one session of the experiment, these participants were not excluded as our use of the hierarchical Bayesian techniques enables a balance between unequal amounts of individual participant data.

#### Materials

The word stimuli were drawn from a word pool consisting of 1608 medium-frequency words, ranging from four to eight letters ($M = 5.88$, $SD = 1.31$), and ranged in word frequency from 10 to 40 counts per million ($M = 19.98$, $SD = 8.06$). Word frequency was sourced from the SUBTLEX corpus (Brysbaert & New, 2009).

The face stimuli were drawn from an online database consisting of AI-generated human faces (Generated Photos: https://generated.photos/). Faces from four ethnicities and two genders were selected, such that there were 148 Asian females, 112 Asian males, 134 African females, 168 African males, 143 Latinx females, 180 Latinx males, 117 European females, and 149 European males. The selected photos were all front-facing adult faces with joy expressions and a white background (available in our OSF repository https://osf.io/au94s).

The words and faces were tested in different pure list conditions, such that in each list all stimuli were either words or faces. For each participant, a total of two eight-word lists and sixteen 24-word lists were drawn pseudo-randomly without replacement from the word pool. Similarly, a total of two eight-face lists and sixteen 24-face lists were drawn pseudo-randomly without replacement from the selected face pool, with each ethnicity-gender group being equally represented in each list. Half of the ten-item and 40-item lists were designated to be the practice and experimental study lists, respectively, while the remaining lists served as lures in the practice test/test lists.

#### Procedure

The experiment was coded using the jsPsych package in JavaScript (de Leeuw, 2015). Each participant completed the experiment through a unique link on their own devices. A minimum of 24-h break was enforced between sessions to prevent fatigue. A within-subjects design was adopted where participants experienced two types of stimuli – words or faces. The order of the conditions was randomized, with the nature of the stimuli being informed immediately prior to the study lists.

The experiment consisted of two sessions, with each session lasting approximately 45 min. During each session, participants were to complete a response-key practice block, a practice and four experimental blocks of computer-based recognition memory tasks.

To encourage participants to use all of the confidence ratings, we included a response key practice block where participants were told that they would be presented with a series of confidence options (‘def. old, ‘prob. old’, ‘maybe old’, ‘maybe new’, ‘prob. new’, and ‘def. new’) in capital letters one at a time, and their task was to respond as quickly and accurately as possible using corresponding keys. Participants were instructed to place their left-hand ring, middle, and index fingers on the S, D, and F keys and their right-hand index, middle, and ring fingers on the J, K, and L keys. The order of the confidence options was randomized within subjects such that on some sessions the S, D, F, J, K, and L keys corresponded to options from ‘def. old’ to ‘def. new’, respectively, while on some sessions the keys corresponded to options from ‘def. new’ to ‘def. old’, respectively. Feedback of ‘CORRECT!’ or ‘WRONG!’ were given on the screen for 800 ms for correct/incorrect responses, while a ‘TOO SLOW! RESPOND FASTER!’ message will appear if participants did not give a response within 1500 ms. Each of the six confidence options were repeated five times, resulting in 30 trials in total. The repetition, however, was conducted pseudo-randomly in which immediate repeated presentation was not allowed.

Each practice and experimental block consisted of two study-test cycles, with each cycle corresponding to different stimulus types (i.e., words vs. faces). Each experimental cycle consisted of a study phase, distractor phase, and test phase. The practice task did not include a distractor phase. During the study phase, a list of items flashed on the computer screen, one at a time. For word stimuli, the presentation rate was 750 ms per item followed by a 150-ms interstimulus interval, whereas each face stimulus had a longer presentation time of 1750 ms followed by a 150-ms interstimulus interval to increase performance.

**Fig. 3:**
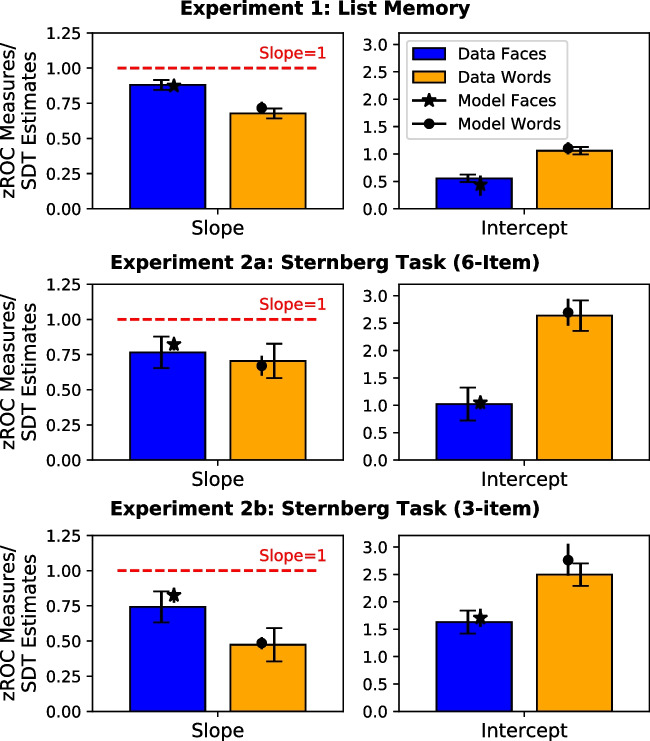
Mean zROC slopes and intercepts along with posterior means of the $\sigma _{lure}$ and $\mu _{target}$ for words and faces from all experiments. *Note.* For mean slopes and intercepts, error bars represent 95% within-subjects confidence intervals. For posterior means, error bars represent 95% highest density intervals

Immediately after the study phase, a message appeared prompting participants to proceed to a simple true/false mathematical task for 45,000 ms. Each of the math problems was displayed in the form of A + B + C = D, where A, B, C, D were numbers. Participants were asked to determine whether D was the true sum of the numbers on the left of the equation by pressing corresponding keys ("1" for TRUE and "0" for FALSE).

Following this, participants were directed to begin the test phase in which they were asked to respond whether each test item had appeared in the study lists or not. Meanwhile, they had to state their level of confidence in this recollection using a six-option confidence rating scale (i.e., three levels of confidence for two choices - old and new). Quick and accurate responses were encouraged – if a response was given between 280 and 8000 ms, no message would appear; whereas if a response was given beyond 8000 ms, a ‘TOO SLOW! RESPOND FASTER!’ message would appear on the screen for 800 ms, while a ‘TOO FAST! THINK CAREFULLY!’ message would appear for responses given below 280 ms.

In order to encourage participants to spread their use of keys among all six confidence options, a token-earning game was implemented during the test phase. Each correct/incorrect response was worth +3/-3 points for high-confidence, +2/-2 points for medium-confidence, and +1/-1 point for low-confidence responses. As high-confidence keys were associated with higher penalty, the game should in principle motivate participants into strategically using low-confidence response keys when lacking evidence and less assured.

### Results

Data from 13 participants were excluded, for displaying bad task performance or non-adherence to the instructions (see Supplementary Material in our OSF repository https://osf.io/au94s). Responses with reaction times (RTs) less than 300 ms and greater than 4000 ms were excluded as these were likely to be guesses, resulting in a loss of 1.55% of total data. The raw data can be found in our OSF repository (https://osf.io/au94s).

#### Empirical analysis

To quantify the amount of evidence for or against an effect, Bayesian hypothesis tests were performed using JASP (Team, 2020). The Bayes factor presents a comparison between two competing hypotheses (i.e., null and alternative), with its value quantifying the updates in belief from the data for one of the hypotheses (Wagenmakers et al., 2018). A Bayes factor (i.e., $\text {BF}_{10}$) larger than 1 indicates evidence for an effect, whereas a Bayes factor smaller than 1 suggests evidence for absence of an effect. According to Lee and Wagenmakers (2013), $1<\text {BF}<3$ is considered as inconclusive/anecdotal evidence; $3\le \text {BF}<10$ as moderate evidence; $10\le \text {BF}<30$ as strong evidence; and $30\le \text {BF}<100$ as extreme evidence.

The slopes and intercepts of words and faces zROCs for all experiments can be seen in Fig. 3. For simplicity, the slope values were directly derived from the observed zROC curves by applying linear least-squares regressions (i.e., the raw slopes). A set of Bayesian one-sample *t* test (Jeffreys, 1961) were performed to investigate whether target variance was smaller than lure variance. There was extreme evidence suggesting that the zROC slopes for words ($M = 0.68, SD = 0.21$) and faces ($M = 0.88, SD = 0.14$) were both smaller than 1.0, $t(66) = -12.75, \text {BF}_{10} = 5.74{\hspace{-1.111pt}\times \hspace{-1.111pt}}10^{+16}$ for words and $t(66) = -6.92, \text {BF}_{10} = 9.86{\hspace{-1.111pt}\times \hspace{-1.111pt}}10^{+6}$ for faces. To investigate whether our manipulation on stimulus types did induce differences in zROCs and performance, a series of Bayesian one-way within-subjects analyses of variance (ANOVAs; Morey & Rouder, 2015; Rouder, Morey, Speckman, & Province, 2012) were performed for each dependent variable of interest. Extreme evidence was found for the slope to be smaller for words than for faces, $F(1, 66) = 66.25, \text {BF}_{10} = 3.32{\hspace{-1.111pt}\times \hspace{-1.111pt}}10^{+9}$, while intercept was larger for words than for faces, $F(1, 66) = 108.60, \text {BF}_{10} = 1.55{\hspace{-1.111pt}\times \hspace{-1.111pt}}10^{+13}$.

#### SDT modeling

We complemented the analyses above by fitting the SDT model to individual participant ROC data using hierarchical Bayesian techniques (see Rouder & Lu, 2005, for an introduction). A major advantage of this method is that hierarchical Bayesian models are resistant to noise and outliers – as the group-level and the individual participant-level parameters are separately estimated, extreme values of parameters are pulled toward the group estimates (a phenomenon termed ‘shrinkage’). This is advantageous, considering that the SD parameters of SDT models are often difficult to estimate.

As the words and faces were tested in different pure list conditions (e.g., all stimuli were either words or faces), parameters were separately estimated for each condition with no shared information between them: (1) the SDs of the lure distributions; (2) the means of the target distributions; and (3) the confidence criteria (five criteria needed for six confidence ratings). Note that as different confidence criteria were applied to words and faces, there were ten criteria estimated in total. The SDs of the target distributions were fixed to 1.0, such that the ratio of the lure-to-target variability ($\sigma _{lure}/\sigma _{target}$) can be directly derived from the SDs of lures. The means of the lure distributions were fixed to 0.0 to identify the model.

Minimally informative prior distributions were applied to impose mild constraints on the parameter values. For estimating the posterior distribution, a typical approach is to use the Markov chain Monte Carlo (MCMC) algorithm. Yet, considering the possible challenges from correlated parameter estimates in SDT models, the differential evolution MCMC (DE-MCMC: Turner, Sederberg, Brown, & Steyvers, 2013), which is a posterior sampling method that is more robust to parameter correlations, was instead employed. Details of the prior distributions and DE-MCMC procedure are provided in the Appendix. Model codes are available in our OSF repository (https://osf.io/au94s).

To verify whether unequal variance and specifically greater target variability was indeed favored, model selection was performed, which compared the equal-variance SDT with three versions of unequal-variance SDT – one with $\sigma _{lure}$ being freely estimated; one with $\sigma _{lure}$ constrained to be larger than 1.0; and one with $\sigma _{lure}$ constrained to be between 0.0 and 1.0. As models can vary in their complexity and therefore capability to account for data, the Widely Applicable Information Criterion (WAIC: Watanabe , 2010) was adopted for model selection for its ability to take into account the trade-off between complexity and goodness of fit. A more complex model receives harsher penalty, such that for it to be preferred, larger improvement in fit is needed to outweigh the penalty. To facilitate model comparison, we report the WAIC difference scores, in which the winning model has a score of zero while all other models have positive values reflecting the differences in WAIC between them and the best model. The model selection results for all experiments are presented in Table 1. By convention, WAIC differences of ten or more between models would be considered ‘large’. Model selection in Experiment 1 favored the unequal-variance SDT with freely estimated $\sigma _{lure}$, indicating no support for equal variability between targets and lures.

**Table 1:** WAIC difference scores for EVSD and three versions of UVSD from all experiments

	Exp1		Exp2a		Exp2b	
Model	Faces	Words	Faces	Words	Faces	Words
EVSD^a^	310	762	106	44	59	75
UVSD^b^	**0**	**0**	2	**0**	**0**	1
UVSD + Greater target variability^c^	5	10	**0**	1	4	**0**
UVSD + Greater lure variability^d^	323	767	121	57	71	83

*Note.* The winning model is depicted in bold. ^a^SDT model with equal variance. ^b^SDT model with unequal variance. ^c^SDT model with unequal variance and $\sigma _{lure}$ constrained to be smaller than 1. ^d^SDT model with unequal variance and $\sigma _{lure}$ constrained to be larger than 1

**Fig. 4:**
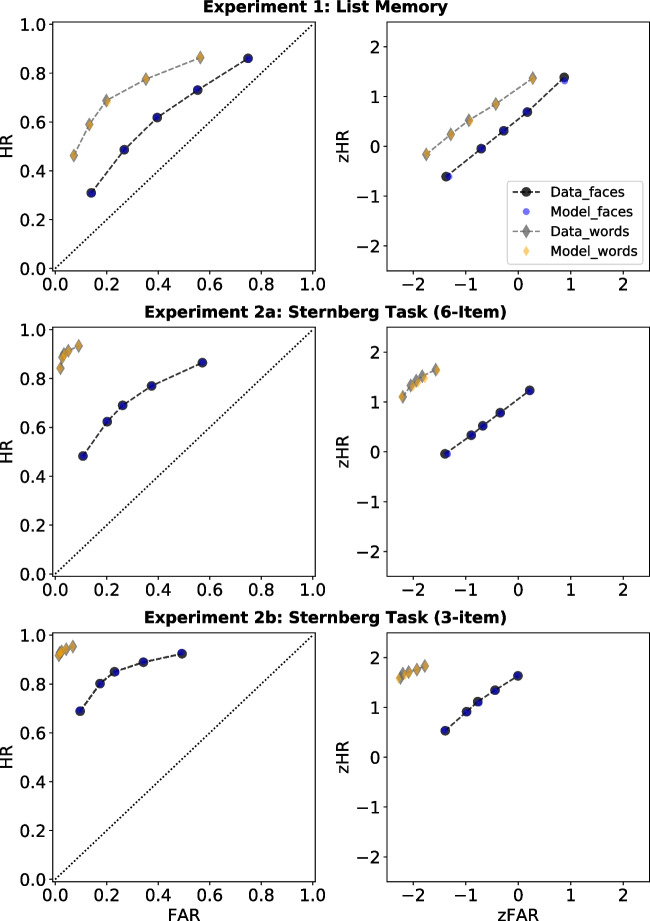
Estimated and observed ROCs (*left*) and zROCs (*right*) for words and faces in all experiments. *Note.* *Black dots* and *grey diamonds joined by dotted lines* represent observed data from the faces and words conditions, respectively. *Blue dots* and *orange diamonds* represent SDT model predictions for faces and words, respectively

Figure 4 shows the observed ROCs (left panel) and zROCs (right panel) along with the predicted curves by the best-fitting SDT model to the data, for all experiments. The grey diamonds and black circles joined by the dotted lines represent the data; the orange diamonds and blue circles represent the SDT model predictions, both for words and faces respectively. From visual inspection, the parallel zROCs of Experiment 1 (top right panel) do not differ systematically from linearity.

The SDT model correctly captured the curvilinear shape of the ROCs and the linear shape of zROCs in both conditions of stimulus type. The SD ratios generated by the best-fitting models for words and faces across all experiments can be seen in Fig. 3. As expected, the mean SD ratio for words ($\sigma _{lure} = 0.72$) and faces ($\sigma _{lure} = 0.87$) were slightly different than the observed slopes but again smaller than 1.0, with the 95% highest density intervals (HDIs) indicating clear differences to 1.0. The slight deviation between model parameter estimates and the empirical zROC measures reflects a correction by using the hierarchical Bayesian techniques in the presence of noise in the data.

### Discussion

We found zROC slopes that were smaller than 1.0 in both the condition that used words and faces as stimuli. This remained true after corrections achieved by conducting the hierarchical Bayesian analyses. Despite a clear difference in performance between words and faces, a reversal of the usual pattern of greater target variability was not observed. Taken together, these results indicate that the use of face stimuli may not be responsible for the observation of greater variability in lure stimuli found in eyewitness memory paradigms.

## Experiment 2a & 2b

In addition to the use of non-linguistic stimuli, the tasks in Yotsumoto et al. (2008) only involved short-term memory retrieval when study lists of only three items and single test probe were employed. An additional possibility is thus suggested that greater variability of lure stimuli may be more likely to be produced under conditions with short-term memory retrieval. To facilitate comparison with previous studies, a short-term Sternberg paradigm (Sternberg, 1966) was adopted for Experiment 2a and 2b. Such a paradigm was chosen not only for a partial replication of Yotsumoto et al. (2008) but also for their unique property shared with eyewitness identification, that only one identification decision is made during test. Experiment 2a utilized study lists of six items while Experiment 2b used a shorter list of three items. To prevent ceiling level performance, we adopted shorter presentation times for conditions of higher performance (shorter lists and word stimuli).

A common finding in the Sternberg paradigm is better performance for more recent items (e.g., Kahana & Sekuler, 2002; Monsell , 1978; Osth et al. , 2023; Nosofsky, Little, Donkin, & Fific, 2011). Thus, we additionally analyzed whether zROC slope varies systematically across serial positions for both stimulus types.

### Method

#### Participants

A total of 33 and 29 participants contributed to Experiment 2a and 2b, respectively. Despite the smaller sample sizes as compared to Experiment 1, participants in Experiment 2a and 2b were expected to complete three 1-h sessions of the experiments. This in total gave us 336 (Experiment 2a) and 648 (Experiment 2b) trials per condition for each participant (if they completed all sessions), which were comparable to 144 and 800 trials each participant was tested on in studies with similar Sternberg-styled design (Sternberg, 1966; Yotsumoto et al., 2008). In Experiment 2a, two participants did not complete the final session, while in Experiment 2b, only 14 participants managed to complete all three sessions.

#### Material

The word and face stimuli were drawn from the same word/face pool as the first experiment. For Experiment 2a, a total of 113 six-item study lists and 113 lure items were drawn pseudo-randomly without replacement from the word pool, with the same number of lists/items drawn from the face pool. For Experiment 2b, a total of 217 three-item study lists and 217 lure items were drawn pseudo-randomly without replacement from the word pool, with the same number of lists/items drawn from the face pool. For both experiments, one list and one lure item were randomly selected from each stimulus type to serve at the practice stage, with the remaining served at the actual experiment. Unlike Experiment 1, each list of faces (and the corresponding lures) was focused on a different ethnicity-race group. Yet, the eight ethnicity-race groups were still equally represented, such that in the actual experiment, each group occupied an equal number of lists.

#### Procedure

Experiment 2a and 2b both consisted of three sessions, with each lasting approximately 45 min. In each session, Experiment 2a had one response-key practice block, one practice, and 112 experimental blocks of computer-based recognition memory tasks; whereas Experiment 2b had one response-key practice block, one practice, and 216 experimental blocks.

Each practice and experimental block consisted of two study-test cycles, with each cycle corresponding to different stimulus types (i.e., words vs. faces). During each cycle, a fixation cross was first presented on the screen for 1000 ms. This is followed by presentations of six study items one at a time (each presented for 500 ms if it was a word, and 1250 ms if it was a face), separated by a 150-ms inter-stimulus interval. After presentation of the study lists, a 1000 ms fixation cross appeared again, followed by a single test item. During each test trial, a countdown timer was displayed at the top of the screen, indicating that amount of time left before the trial ended. Participants were to identify whether the test item was one of the study items or not, using a six-option confidence rating scale as stated in Experiment 1. The probability of the test item being a target or lure was equally distributed (i.e., 50% for being a target and 50% for being a lure). Serial positions were also controlled, in that there was a roughly equal number of targets from each serial position. Again, the too-slow and too-fast feedback was provided if participants responded slower than 8000 ms or faster than 280 ms. In the practice block, additional feedback on correct/incorrect responses was provided. Also, the token earning game was again employed to encourage use of all response keys.

**Fig. 5:**
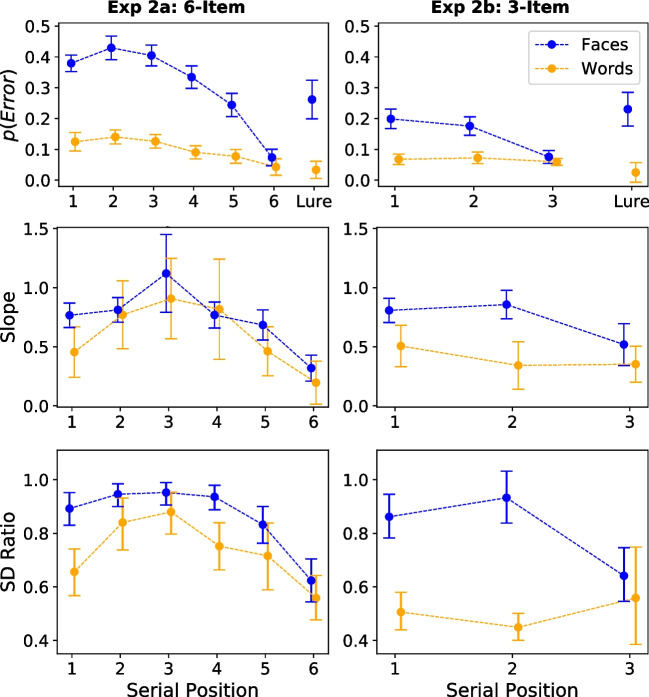
Error rates, mean zROC slopes and posterior means of $\sigma _{lure}$ for words and faces across serial positions in Experiment 2a and 2b. *Note.* The *top* and *middle panels* depict the error rates across study positions and lures, along with mean zROC slopes across study positions from Experiment 2a and 2b. *Error bars* represent 95% within-subjects confidence interval. The bottom panel shows the posterior means of the $\sigma _{lure}$ for words and faces across serial positions from Experiment 2a and 2b. *Error bars* represent 95% highest density intervals

The procedure of Experiment 2b was mostly identical to that of Experiment 2a, but with a couple of exceptions. Firstly, participants were only presented with three study items during the study phase. Secondly, to prevent performance at ceiling level due to shorter lists, presentation times were shortened such that each word was presented for 250 ms and each face was presented for 750 ms, followed by a shorter interstimulus interval of 75 ms. Finally, serial positions were better controlled as there was a exactly equal number of targets from each position.

### Results

Data from four participants were excluded for either displaying bad task performance or non-adherence to the instructions (two were from Experiment 2a, two were from Experiment 2b; see Supplementary Material). For some participants, the zROC slopes and intercepts were unable to obtain and therefore marked as missing values when performing ANOVAs and *t* tests. These participants possessed straight vertical lines for zROCs as they only had one data point on the *x*-axis (the FARs). The vertical zROCs were not caused by bad performance, instead, these were due to high performance in short lists where participants tended to use only the high-confidence keys, thus resulting in insufficient ROC points used to calculate slopes and intercepts (see Supplementary Material). Responses with RTs less than 300 ms and greater than 4000 ms were excluded, resulting in losses of 3.27% and 1.89% of total data in Experiment 2a and 2b. The raw data can be found in our OSF repository (https://osf.io/au94s).

#### Empirical analysis

The ROCs and zROCs for Experiment 2a and 2b are displayed in the middle and bottom panels of Fig. 4. From visual inspection, the zROCs for faces do not differ systematically from linearity, whereas the zROCs for words deviate mildly from linearity. The zROC slopes for words ($M = 0.71, SD = 0.52$ for Experiment 2a; $M = 0.47, SD = 0.42$ for Experiment 2b) and faces ($M = 0.75, SD = 0.16; M = 0.77, SD = 0.17$) in both experiments were smaller than those from Experiment 1 in most cases, except the slope for words in Experiment 2a. Again, all slopes across stimulus types and experiments were smaller than 1.0, which was supported by strong and extreme evidence, $t(25) = -2.94, \text {BF}_{10} = 12.69$ for words in Experiment 2a, $t(20) = -5.71, \text {BF}_{10} = 3280.20$ for words in Experiment 2b, $t(30) = -7.75, \text {BF}_{10} = 2.16{\hspace{-1.111pt}\times \hspace{-1.111pt}}10^{+6}$ for faces in Experiment 2a, and $t(26) = -5.67, \text {BF}_{10} = 7109.34$ for faces in Experiment 2b.

A series of Bayesian two-way within-subject ANOVAs (as we manipulated both stimulus types and serial positions) were performed for each dependent variable of interest. Anecdotal evidence suggested no effect of stimulus type on slopes in Experiment 2a, $F(1, 125) = 3.50, \text {BF}_{10} = 0.31$, whereas strong evidence was found for slopes being smaller for words than for faces in Experiment 2b, $F(1, 40) = 16.94, \text {BF}_{10} = 23.79$. Very strong and extreme evidence supported intercepts to be increased for words than for faces in the two experiments respectively, $F(1, 125) = 85.99, \text {BF}_{10} = 1.83{\hspace{-1.111pt}\times \hspace{-1.111pt}}10^{+7}$; $F(1, 40) = 42.27$, $\text {BF}_{10} = 3644.34$.

**Table 2:** WAIC difference scores for an equal-variance and three versions of unequal-variance serial-position SDT from experiment 2a and 2b

	Exp2a		Exp2b	
Model	Faces	Words	Faces	Words
EVSD^a^	74	62	72	82
UVSD^b^	1	3	**0**	**0**
UVSD + Greater target variability^c^	**0**	**0**	5	.02
UVSD + Greater lure variability^d^	111	99	95	106

*Note.* The winning model is depicted in bold. ^a^Serial-position SDT model with equal variance. ^b^Serial-position SDT model with unequal variance. ^c^Serial-position SDT model with unequal variance and $\sigma _{target}$ constrained to be larger than 1. ^d^Serial-position SDT model with unequal variance and $\sigma _{target}$ constrained to be smaller than 1

Consistent with previous literature, decreased error rates for items in more recent study positions was observed (see Fig. 5, top panel). This, however, raises one concern that averaging slopes across serial positions may obscure any different patterns occurring among the positions. Consider a case where there could be a reversal of the pattern – if long-term and short-term memory retrieval indeed differ in the direction of evidence variability, with tasks that requires long-term memory retrieval displaying greater target variability while tasks that require short-term memory retrieval displaying greater lure variability, the average of slopes from older serial positions (i.e., likely reflects long-term memory retrieval) and from later serial positions (i.e., short-term memory retrieval) might be pushed more toward greater target variability. A simple way to test this is to obtain zROC slopes for each serial position and see whether the slopes are smaller than 1.0 for any position.

The means and 95% within-subject confidence intervals of the slopes for each serial position from both experiments are displayed in the middle panel of Fig. 5. As suggested by Bayesian *t* test results, most but not all slopes received very strong to extreme evidence for displaying greater target variability ($\text {BF}_{10} = 20287.61; 0.70; 0.30; 0.44; 179.81; 4.38{\hspace{-1.111pt}\times \hspace{-1.111pt}}10^{+10}$ for words, $\text {BF}_{10} = 163.82; 34.96; 0.12; 125.96; 4835.82; 7.99{\hspace{-1.111pt}\times \hspace{-1.111pt}}10^{+10}$ for faces in Experiment 2a; $\text {BF}_{10} = 46.65; 1670.71; 32261.18$ for words, $\text {BF}_{10} = 30.28; 1.45; 31428.58$ for faces in Experiment 2b). However, as there might be insufficient data per serial position, the slope values were vulnerable to noise and outliers in the data. For example, for the only slope that had a mean value greater than 1.0 (i.e., the slope for the third position of faces condition), the group average value was predominantly influenced by an outlier who had a slope of 5.42. The reason why this participant had such a large slope is because he/she had a very tiny variation in FARs ($\text {FAR}_{max} - \text {FAR}_{min} = 0.007$), making it extremely easy for the HRs to have a larger variation than that of the FARs, which resulted in an exceptionally large slope. Excluding this outlier resulted in an average slope of 0.98 for the third serial potion in faces condition. These issues thus motivated further investigation using hierarchical Bayesian analyses.

#### SDT modeling

A equal-variance and three versions of unequal-variance SDT models were again fitted to check whether overall, the target variance exceeded lure variance in both words and faces. No preference for the equal-variability or greater lure variability model was found in model selections of both experiments (see Table 1). The predicted ROCs and zROCs generated by the best-fitting model are displayed in the middle and bottom panels of Fig. 4 for Experiment 2a and 2b, respectively. The best-fitting SDT models fitted well to the curvilinear ROCs in words and faces as well as the linear zROCs for faces in both experiments. However, it failed to capture the nonlinear zROCs of words in either experiment. The SD ratios again differed slightly compared to the observed slope values ($1/\sigma _{target} = 0.66$ for words and $1/\sigma _{target} = 0.81$ for faces in Experiment 2a; $1/\sigma _{target} = 0.48$ for words and $1/\sigma _{target} = 0.83$ for faces in Experiment 2b). The SD ratios for words were smaller than that for faces, but again all smaller than 1.0, as indicated by the 95% HDIs in the middle and bottom panels of Fig. 3. Improved performance for words ($\mu _{target} = 2.68$ for Experiment 2a; $\mu _{target} = 2.76$ for Experiment 2b) than for faces ($\mu _{target} = 1.05; \mu _{target} = 1.71$) was again demonstrated by the differences between distribution means (i.e., *d’*). Better task performance was also observed as compared to Experiment 1, which was reflected in SD ratios further away from 1.0 and larger separation between target and lure distributions.

Following the empirical analysis, we fitted another version of SDT that allowed the SD and mean parameters of the target distribution to vary across serial positions (hereinafter referred to as the serial-position SDT model). The means and SDs of the lure distributions were fixed to 0.0 and 1.0, respectively. Note that the confidence criteria were not separately estimated for each serial position. Model codes are available in our OSF repository (https://osf.io/au94s). Again, an equal-variance version and three unequal-variance versions of the serial-position SDT were fitted to the data. Parameter estimates of the best-fitting model would demonstrate whether serial position effect as well as greater target variability for all serial positions were found.

**Fig. 6:**
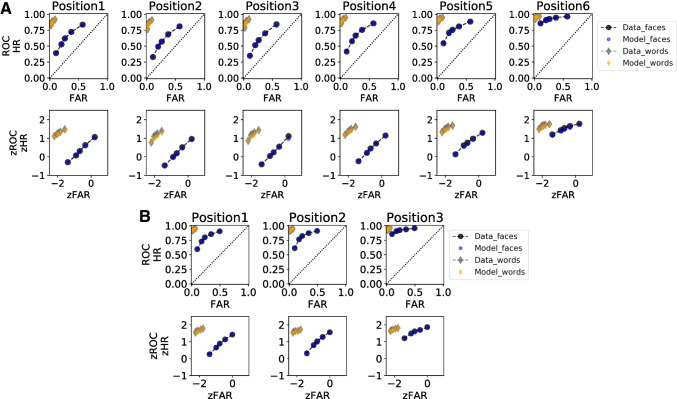
The predicted and observed ROCs and zROCs for words and faces across serial positions in Experiment 2a (**A**) and 2b (**B**). *Note.* *Black dots* and *grey diamonds* *joined by dotted lines* represent observed data from the faces and words conditions, respectively. *Blue dots* and *orange diamonds* represent SDT model predictions for faces and words, respectively

Model selection again indicated no preference for equal-variability in both experiments (see Table 2). The observed ROCs and zROCs along with the predicted curves by the best-fitting serial-position SDT models are displayed in Fig. 6A for Experiment 2a and Fig. 6B for Experiment 2b. Separate ROCs and zROCs are plotted for each serial position. Similar to the basic SDT model, the serial-position SDT model fitted well to curvilinear ROCs and linear zROCs in faces, but slightly misfitted the curvilinear zROCs in words. The SD ratios for words and faces across study positions for both experiments are displayed in Fig. 5, bottom panel. As suggested by the 95% HDIs, the SD ratios in most positions were clearly smaller than 1.0, with the only exception in the second study position of the faces conditions in Experiment 2b. Yet, the most important message here is that there was no evidence for greater lure variability for any study position and stimulus type. The serial-position curves again showed some recency and mild primacy effects, although the curve for words in Experiment 2b remained an exception.

## General discussion

Greater target variability has been found in the vast majority of recognition memory studies, which typically used word stimuli and long-term memory paradigms. However, this finding is not guaranteed to generalize to other conditions. Some rare exceptions have come from eyewitness memory and short-term memory literature (Wixted et al., 2018; Wilson et al., 2019; Yotsumoto et al., 2008), in which greater variability of lure stimuli has been reported. A possibility is therefore suggested that either using non-linguistic stimuli or short-term memory tasks might be responsible for the reversals. The present investigation aimed to evaluate whether either of these conditions would result in greater lure variability.

However, in the current study, greater variability in the target stimuli was found in all conditions and experiments. In Experiment 1 where words and faces were compared within a study-test long-term memory paradigm, zROC slopes smaller than 1.0 were found in both types of stimulus, indicating no reversal of the usual pattern when using non-linguistic stimuli. In Experiment 2a and 2b, where short-term Sternberg tasks were adopted, zROC slopes larger than 1.0 were again not found, regardless of stimulus types. Such findings were also held across serial positions in both experiments (despite one exception in Exp. 2b, evidence for greater lure variability was however never suggested). These results further confirmed that neither short-term memory retrieval nor the use of non-linguistic stimuli were associated with the observation of lure variance exceeding that of targets.

The results that the short-term memory paradigm yielded similar ROC data to those from long-term memory recognition tasks were consistent with a recent study where short-term memory performance was tested using an n-back task and found zROC slopes less than 1.0. Such a finding not only has implications for factors affecting the variability ratio, but also blurs the distinction between short- and long-term memory. A number of researchers have suggested a single store model of memory, which would similarly claim that the same asymmetry in zROC slopes should be observed in both short- and long-term recognition (e.g., Howard & Kahana, 1999; Brown, Neath, & Chater, 2007; Sederberg, Howard, & Kahana, 2008; Greene , 1986; Surprenant & Neath, 2009).

It is worth noting that the variation in the SD parameters across serial positions was large in Experiment 2a and 2b, with target variance increased for initially studied and more recent items. This potentially adds to a growing list of variables that do affect target item variance (Spanton & Berry, 2022), with further research needed to validate such findings. One explanation for such variation is that discriminability has been found to be significantly positively correlated with target variance, in contrast to the constancy-of-slopes hypothesis (Spanton & Berry, 2022). Thus, conditions of improved memory performance would be expected to show lower zROC slopes. In the data from Experiment 2, we found exactly this pattern, as more recent serial positions, which show greatly improved performance, also show considerably reduced zROC slopes. The positive relationship between discriminability and target variability is also naturally predicted by global matching models like Minerva 2 and REM. The same reasoning applies when considering the more uneven variability ratio for words than for faces (see Fig. 3), as face stimuli are usually less discriminable than words. One might also note that while previous research reported U-shaped zROCs for non-linguistic stimuli such as travel scenes (e.g., Onyper et al. , 2010; Howard et al. , 2006), the current results with linear zROCs for faces do not seem to uphold such findings.

The reason why the finding of greater target variability is of particular theoretical importance is because it explains the stronger confidence–accuracy relationship for ‘old’ responses than ‘new’ responses. Namely, greater accuracy has been found to be associated with higher confidence, and this is especially true for *old* responses (Mickes, Wixted, & Wais, 2007). Greater target variability also potentially explains the poorer resolution of confidence for negative decisions (i.e., non-choosers) than positive decision (i.e., choosers) observed in eyewitness identification literature (e.g., N. Weber & N. Brewer, 2004; N. Weber & N. Brewer, 2006).

The SDT framework is typically used to explain how a stronger confidence–accuracy relationship in ‘old’ responses can be accounted for by greater target variability. Referring back to the illustration of SDT in Fig. 1, in order to account for confidence responses, additional criteria are added to partition the distributions into more bins, with each bin corresponding to each confidence option. The accuracy for each confidence option is thus determined by the relative proportion of area under each distribution curve within the corresponding bin. Increasing the variance of the target distribution results in a greater target area for the high-confidence old response bin, producing a stronger relationship between confidence and accuracy.

However, it is not proposed here that previous observations of greater lure variability were due to chance. Instead, it may be that there may be something else specific to the eyewitness identification and short-term memory paradigms that is responsible for the discrepancy. For instance, it is possible that the lineup procedure itself produces such findings. In the eyewitness memory paradigm, there is typically a single study item (i.e., the suspect) along with a test list consisting of one target (i.e., the suspect) and usually five lures (i.e., the fillers). The present study did not closely replicate the eyewitness identification lineups but used Sternberg-styled tasks as an approximation. It thus remains possible that the origin of difference may lie in the procedures that were not manipulated by our experiments.

A methodological explanation for the absence of the expected asymmetry (i.e., greater target variability) in lineup procedures is as follows: while the same innocent or guilty suspect is viewed by all once-tested participants, the fillers are randomly drawn from a large pool of photos that match the description of the suspect. Thus, only the fillers (i.e., the lures) but not the suspects (i.e., the targets) are tested with different items, thus potentially allowing stimulus variability for lures to exceed that of the targets (Shen, Colloff, Vul, Wilson, & Wixted, 2023). By contrast, in designs where the suspects and the fillers are fixed across participants, item variability is no longer differentially added to each distribution and an equal-variance model typically fits the best (Wixted et al., 2018; Shen et al., 2023). It therefore seems plausible that the random selection process might be what contributes to the greater variance of lures in eyewitness paradigms. Nonetheless, it remains an open question as to why there are no cases of greater target variability in eyewitness memory paradigms. In addition, many of the existing explanations – such as the encoding variability hypothesis, as well as the global matching models such as Minerva 2 and REM – would still predict greater target variability even with a single study item, since it is assumed that target variance is induced by a range of variables that affect encoding strength during the study phase.

Alternatively, it is possible that high similarity in items is responsible for the observation of greater lure variability. In the current study, lure items only matched the target items on some basic characteristics (i.e., word frequency for words; race, gender, and age for faces). This is in contrast to eyewitness lineups that usually involve fillers that physically resembles the suspect, and to Yotsumoto et al. (2008) where even the targets were perceptually highly similar to each other. It is theoretically possible that having shared features between targets and lures could affect the estimates of lure variability because any variability in the encoding of targets would affect lures in the same manner, reducing the differences in variability between targets and lures. However, it remains unclear as to why greater lure variability would sometimes be observed. Meanwhile, empirically, there have been several studies that investigated the effects of semantic and orthographic similarity of lures to targets on word recognition memory (Ratcliff et al., 1994; Heathcote, 2003; Neely & Tse, 2009; Cho & Neely, 2013; Dopkins, Varner, & Hoyer, 2017; Shiffrin, Huber, & Marinelli, 1995). While a tendency for lure variance to increase for similar items was sometimes reported (e.g., Ratcliff et al. , 1994; Heathcote , 2003), lure variance exceeding that of targets was otherwise never found. However, an important caveat here is that lure similarity can vary considerably across materials and studies. This might be especially true when the comparison is made with non-linguistic stimuli such as faces or sinusoidal gratings that are not easily rehearsable as words. It is therefore unclear whether the level of similarity between lures in the aforementioned word recognition studies was sufficient or comparable to the level of similarity in eyewitness paradigms or the study of Yotsumoto et al. (2008). The possibility remains that it was the sufficiently high level of similarity in non-linguistic stimuli that prompted the reversal of the usually observed greater variance of targets.

### Conclusion

While recognition memory studies using words as stimuli along with long-term memory paradigms have found greater variance for the target distribution than for the lure distribution, a couple of exceptions that reported evidence for greater lure variability have come from eyewitness memory paradigms (Wixted et al., 2018; Dunn et al., 2022) and short-term memory tasks (Yotsumoto et al., 2008). Comparing stimulus types (faces vs. words) as well as memory paradigms (long-term list-memory paradigm vs. short-term Sternberg-styled paradigm), we attempted to investigate whether one of these factors was responsible for the observation of greater lure variability. Our results showed that lure variance did not exceed that of targets either for face stimuli or in tasks associated with short-term memory retrieval. Yet, it remains possible that some other manipulations such as the lineup procedure or high lure similarity that were not replicated by our experiments were the origin of the discrepancy.

## Figures and Tables

**Fig. 7:**
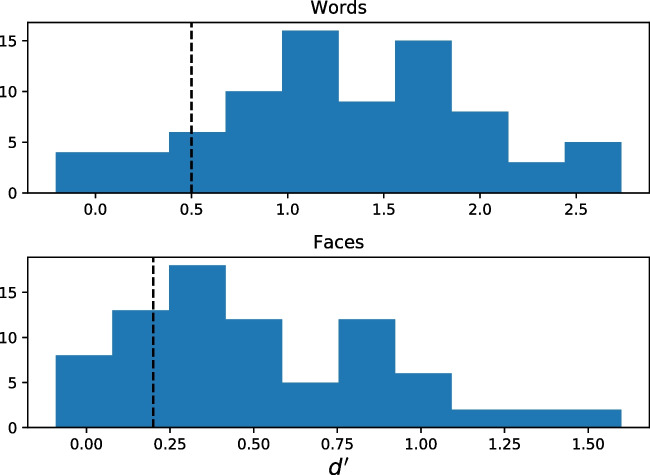
Histogram of participants’ mean discriminability (d’) on words and faces in Experiment 1, *Note.* The top and bottom panel shows participants’ mean *d’* on words and faces respectively, with the black dashed lines representing the 0.5 and 0.2 cutoffs in corresponding condition

**Fig. 8:**
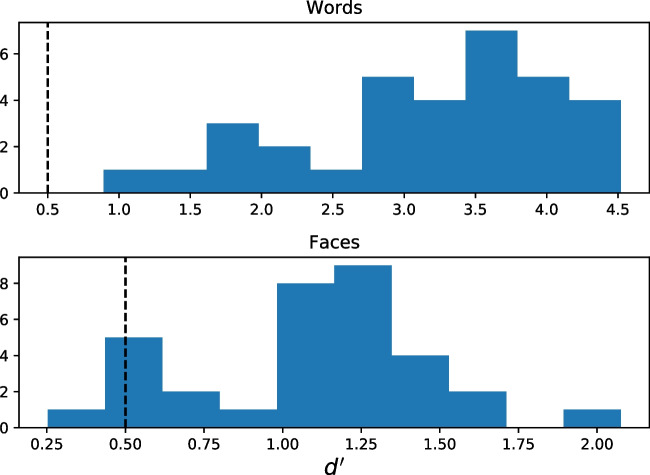
Histogram of participants’ mean discriminability (d’) on words and faces in Experiment 2a, *Note.* The top and bottom panel shows participants’ mean *d’* on words and faces respectively, with the black dashed lines representing the 0.5 and 0.2 cutoffs in corresponding condition

**Fig. 9:**
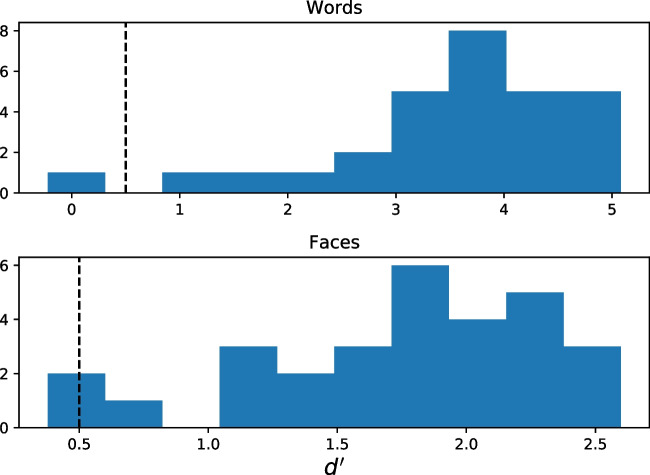
Histogram of participants’ mean discriminability (d’) on words and faces in Experiment 2b, *Note.* The top and bottom panel shows participants’ mean *d’* on words and faces respectively, with the black dashed lines representing the 0.5 and 0.2 cutoffs in corresponding condition

**Fig. 10:**
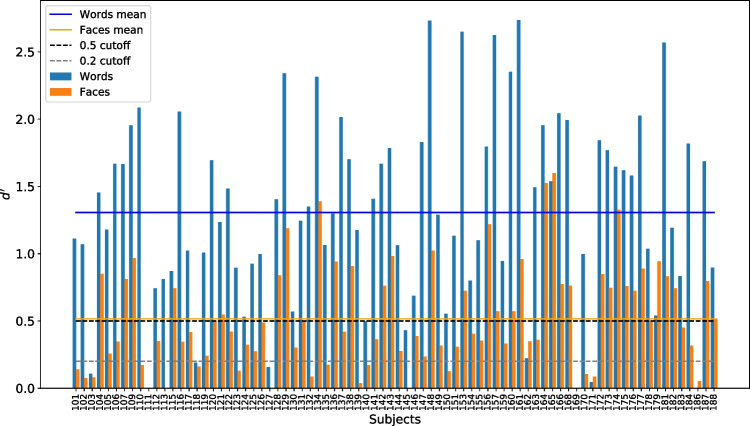
Participants’ mean discriminability (d’) on words and faces in Experiment 1, *Note.* The blue and orange bars represent participants’ mean *d’*, the blue and orange horizontal lines represent the mean *d’* across all participants, the black and grey dashed line represents the 0.5 and 0.2 cutoffs, for words and faces respectively

**Fig. 11:**
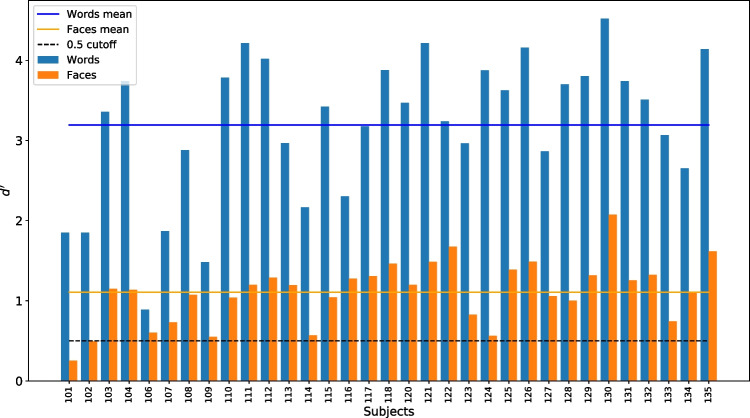
Participants’ mean discriminability (*d’*) on words and faces in Experiment 2a, *Note.* The blue and orange bars represent participants’ mean *d’*, the blue and orange horizontal lines represent the mean *d’* across all participants, the black and grey dashed line represents the 0.5 and 0.2 cutoffs, for words and faces respectively

**Fig. 12:**
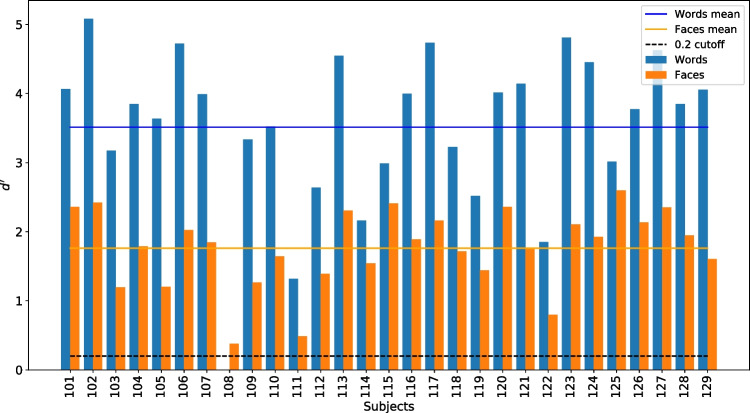
Participants’ mean discriminability (*d’*) on words and faces in Experiment 2b, *Note.* The blue and orange bars represent participants’ mean *d’*, the blue and orange horizontal lines represent the mean *d’* across all participants, the black and grey dashed line represents the 0.5 and 0.2 cutoffs, for words and faces respectively

**Fig. 13:**
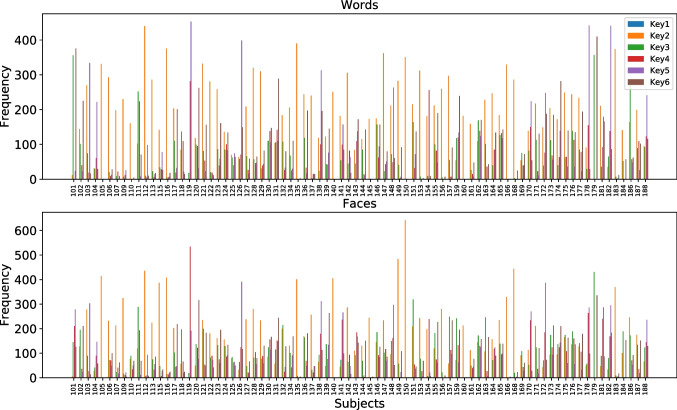
Participants’ usage of the six confidence options for words and faces in Experiment 1,

**Fig. 14:**
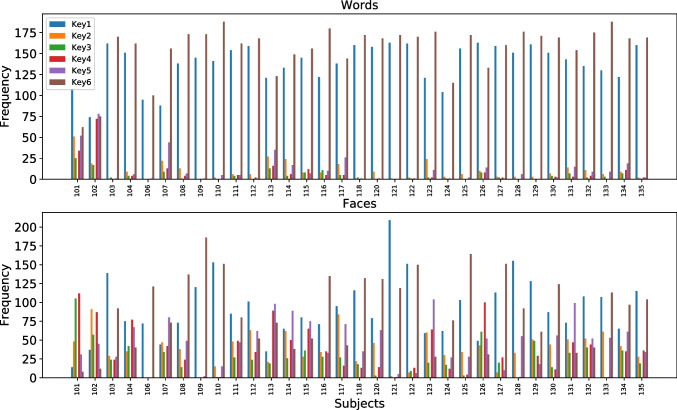
Participants’ usage of the six confidence options for words and faces in Experiment 2a

**Fig. 15:**
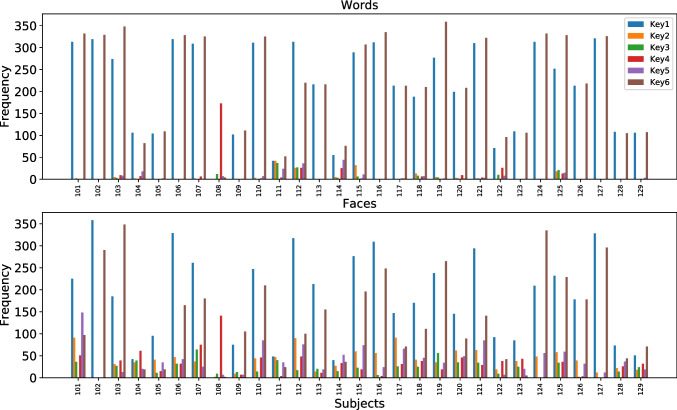
Participants’ usage of the six confidence options for words and faces in Experiment 2b

## Data Availability

The data and materials for all experiments are available on our OSF page (https://osf.io/au94s); none of the experiments was preregistered.

## Appendix

### Prior distributions on model parameters

Participant parameters were sampled from group-level distributions with mean *M* and standard deviation $\varsigma$. Unbounded parameters were sampled from normal distributions, while several bounded parameters were sampled from truncated normal distribution with a lower bound of zero:

1 $$\begin{aligned} \mu \sim TN (M _\mu , \varsigma _\mu , 0, \infty ). \end{aligned}$$

2 $$\begin{aligned} C \sim TN (M _C , \varsigma _C , 0, \infty ). \end{aligned}$$

3 $$\begin{aligned} C _central \sim Normal (M _Ccentral , \varsigma _Ccentral ). \end{aligned}$$

4 $$\begin{aligned} \sigma _t \sim Normal (M _{\sigma t }, \varsigma _{\sigma t }). \end{aligned}$$

5 $$\begin{aligned} \sigma _l \sim Normal (M _{\sigma l }, \varsigma _{\sigma l }). \end{aligned}$$

6 $$\begin{aligned} \sigma \sim TN (M _{\sigma }, \varsigma _{\sigma }, 0, \infty ). \end{aligned}$$

7 $$\begin{aligned} \sigma _st \sim Normal (M _{\sigma st }, \varsigma _{\sigma st }). \end{aligned}$$

8 $$\begin{aligned} \sigma _sl \sim Normal (M _{\sigma sl }, \varsigma _{\sigma sl }). \end{aligned}$$

9 $$\begin{aligned} \sigma _s \sim TN (M _{\sigma s }, \varsigma _{\sigma s }, 0, \infty ). \end{aligned}$$

Minimally informative prior distributions were imposed for all group parameters:

10 $$\begin{aligned} M _{\mu target } \sim TN (1.5, 1.5, 0, \infty ). \end{aligned}$$

11 $$\begin{aligned} M _{\mu lure } \sim TN (0, 1, 0, \infty ). \end{aligned}$$

12 $$\begin{aligned} M _C \sim TN (0.5, 1, 0, \infty ). \end{aligned}$$

13 $$\begin{aligned} M _Ccentral \sim Normal (0, 1). \end{aligned}$$

14 $$\begin{aligned} M _{\sigma }, M _{\sigma s } \sim TN (1, 1, 0, \infty ). \end{aligned}$$

15 $$\begin{aligned} M _{\sigma t }, M _{\sigma l }, M _{\sigma st }, M _{\sigma sl } \sim TN (0, 1). \end{aligned}$$

### Details on MCMC estimation procedure

For each model, the number of chains was set equal to three times the number of participant parameters. To reduce auto-correlation, chains were heavily thinned such that only one in every 20 MCMC iterations was recorded. This process occurred after 5000 burn-in iterations were discarded and continued until 1000 samples were collected.

For a model to be considered converged, its Gelman–Rubin (GR) statistic should be below 1.20 for all parameters.

## Excluson Criteria

A list of exclusion criteria were shared between Experiment 1, 2a and 2b, covering both task performance and adherence to instructions.

### Task Performance

Participants who were likely not paying attention to the tasks were candidates for exclusion. Histograms of the performance measure *d’* from all participants are displayed in Figs. 7, 8 and 9. Cutoffs of *d’* were separately evaluated across conditions and experiments. In Experiment 1, as word stimuli are generally easier for recognition, participants who displayed *d’*< 0.2 in faces as well as *d’*< 0.5 in words were excluded. Participants who displayed *d’*< 0.2 in faces but *d’*>= 0.5 in words were not excluded as they were at least trying at the tasks. In experiment 2a and 2b, as the overall performance was more ideal, participants who displayed *d’*< 0.5 in any one of the conditions were excluded.

As a results, eight participants in Experiment 1 (subject 103, 111, 118, 127, 145, 169, 171, and 186: see Fig. 10) ; one participant in Experiment 2a (subject 101: see Fig. 11); and two participants in Experiment 2b (subject 108 and 111: see Fig. 12) were excluded.

### Adherence to Instructions

Participants who only used one response key within a 6-option confidence rating scale did not adhere to the instructions and were therefore excluded. As a result, five participants in Experiment 1 (subject 105, 140, 150, 160 and 166: see Fig. 13); one participant in Experiment 2a (subject 106: see Fig. 14) were further excluded, while none of the participants was excluded from Experiment 2b (see Fig. 15).
